# Acceptability of Pulse-Fortified Foods by Two Groups: Participants in a Clinical Trial and Participants in a Consumer Acceptability Panel

**DOI:** 10.3390/foods7080129

**Published:** 2018-08-18

**Authors:** Donna Ryland, Peter Zahradka, Carla G. Taylor, Rhonda C. Bell, Michel Aliani

**Affiliations:** 1 Department of Food and Human Nutritional Sciences, University of Manitoba, Winnipeg, MB R3T 2N2, Canada; Donna.Ryland@umanitoba.ca (D.R.); PZahradka@sbrc.ca (P.Z.); Carla.Taylor@umanitoba.ca (C.G.T.); 2 Department of Physiology & Pathophysiology, University of Manitoba, Winnipeg, MB R3E 3P5, Canada; 3 The Canadian Centre for Agri-Food Research in Health and Medicine (CCARM), St., Boniface Hospital Albrechtsen Research Centre, 351 Tache Avenue, Winnipeg, MB R2H 2A6, Canada; 4 Department of Agricultural, Food and Nutritional Science, University of Alberta, Edmonton, AB T6G 2E1, Canada; bellr@ualberta.ca

**Keywords:** acceptability, pulse-fortified foods, clinical trial, peas, beans

## Abstract

Pulses are nutrient-rich ingredients used as interventions in clinical trials to determine their effect on lowering blood lipids, which are risk factors for cardiovascular disease. Acceptability of these foods is critical for compliance by participants in clinical trials as well as regular consumption by those eating them for their health benefit. Commercialisation of foods that prove positive for health is required to make them available to the general population. Since the target for commercialisation would be products that will be procured by as many people as possible, the research question becomes whether or not testing is required by the clinical trial participants, by consumer acceptability testing in a sensory unit, or by both to ensure acceptability. The objective of this study was to determine the acceptability of pulse-based soups and casseroles destined for a clinical trial by both the participants in the clinical trial and by consumer participants not in the clinical trial. Neither group received any training regarding sensory analysis. Acceptability of aroma, appearance, flavor, texture, overall acceptability, and the frequency of eating the samples of five formulations fortified with either peas or beans was measured. Groups differed in their acceptability of foods for different attributes with the clinical trial participants providing less discrimination among the sensory attributes for their acceptability. Influential factors could include motivation for healthy eating, age, number of times the product was consumed, amount of the product consumed, and where it was consumed. In conclusion, acceptance measures from both groups are required in order to gain as much information as possible regarding acceptability of attributes for commercialisation of pulse-fortified foods that provide a health benefit.

## 1. Introduction

Pulses refer to dried seeds coming from the legume family that are low in fat [1]. They provide a good source of fibre, protein, folate, and minerals including iron, calcium, and potassium [2] which contribute to a healthy diet. The soluble and insoluble fibre present in pulses plays an important role in reducing age-related diseases [3]. Health benefits of consuming pulses include improvement of lipid profiles [4,5] and lowered blood pressure [6] that can influence cardiovascular disease, reduction of risk factors for metabolic syndrome [7], possible weight reduction [8], positive effects for glycemic control [9,10], and lowered colorectal cancer risk [11].

Meta-analysis of a number of clinical trials incorporating pulses determined that 130 g (about ¾ cup) per day significantly reduced LDL-cholesterol levels compared with pulse-free diets [5], while ½ cup per day over eight weeks was sufficient to improve blood flow to the lower limbs of individuals with peripheral artery disease [12]. Consumption patterns are low with 13% of Canadians eating pulses on any given day [13] and on average 1 cup per week [14]. Frequency of foods containing pulses and or pulse flour eaten at home or at a restaurant, respectively, was as follows: beans—59% and 26%; peas—45% and 17%; lentils—33% and 17%. Dishes made with beans included 68% chili, curries, and stews, 57% soups, and 30% salads. For peas, soups were made most often at 82% followed by main dishes and casseroles at 26%, and chili, curries, and stews at 26%. Soups, curries/chili/stews, and main dishes, e.g., casseroles including lentils, were made 82, 33, and 29% of the time, respectively [14]. Given the evidence that consumption of pulses can improve important indicators of health status, it has been suggested that more convenient and familiar foods should be developed [15]. These products also need to be acceptable in terms of aroma, appearance, flavor, and texture to increase consumption of foods containing pulses at levels that generate positive effects on health. The most often cited reasons for eating pulses included tastes good/I like them, 36%; healthy/good for you, 34%; source of protein, 12%; source of fibre, 11%; part of recipe, 10%; and good for soup/stews, 8% [14].

Acceptability is also important when designing foods for a clinical trial to have high levels of compliance to the study protocol. Participants in a clinical trial conducted over a one-year period scored flax-fortified muffins similar in enjoyment as the non-flax muffins and dropout rates were similar between the two groups at 20 to 25% [16]. Acceptability of soy-fortified muffins was significantly higher compared with wheat muffins according to the participants in another clinical trial [17]. In both studies the fortified products were deemed to reduce LDL-cholesterol and could be possible candidates for mainstream commercialisation. However, whether these products have high enough acceptability for those not concerned with any health issue is not known. Although the food development process for food interventions for a clinical trial has included a consumer acceptability study [18], the clinical trial participants were not surveyed regarding their assessment of food acceptability. Health motivation is one factor that differentiates these two groups (clinical trial participants and regular consumers) regarding the acceptability of fortified foods. Other variables include environment where the food is consumed (home environment in the case of some clinical trials versus laboratory or central location for consumer sensory studies), the amount of food eaten at one time (one serving for clinical trial participants versus a portion of a serving for consumer studies) and the duration that it is eaten (clinical trials for a period of time versus a single time for consumer studies). With respect to foods containing pulses, tolerability was considered acceptable by the participants of one clinical trial where this issue was examined [19].

Ultimately, if foods used in a clinical trial show positive results, commercialisation of the foods would be warranted. Since the target for commercialisation would be to make the product so that as many people as possible would accept it, the question becomes whether or not acceptability testing is required of the clinical trial participants, a consumer acceptability test in a sensory unit, or both.

Therefore, our objective was to determine the acceptability of pulse-fortified foods by two different groups: those eating the foods as participants in a clinical trial and those eating the foods as participants in a consumer acceptability study. This exploratory study will be used to generate hypotheses for future study.

## 2. Materials and Methods

### 2.1. Materials

Given the higher consumption of beans and peas noted above, these pulses were chosen as the intervention for the clinical trial. Four types of beans were included—black, navy, pinto, and great northern—and two types of whole peas—yellow and green. Approximately 25 kg of each pulse type were sourced by Pulse Canada (Winnipeg, MB, Canada). Black, navy, and pinto beans were obtained from AGT Food and Ingredients, St. Joseph, MB, Canada; great northern beans from Viterra Inc., Bow Island, AB, Canada; and yellow (CDC Meadow variety) and green (CDC Sage variety) peas from The Scoular Company, Tisdale, SK. All samples were kept in the dark at 21 °C for the duration of the study.

### 2.2. Samples

#### Cooking

Beans and peas were washed for one minute under cold running water and soaked overnight (~16 to 20 h) at 4 °C with tap water in a ratio of 1 part beans or peas to 3 parts water (*wt*/*wt*). Soaking water was drained and soaked pulses were washed for one minute under cold running water, weighed, and cooked in water in an amount that was three times the soaked weight. Pulses were added to the water and placed into a 10 L stainless steel saucepan. The element range top (Frigidaire Household Electric Range, ES510 Control, Electrolux Canada Corp., Mississauga, ON, Canada) was set at high heat. Once a full boil was achieved, heat was reduced to maintain the simmer state and heating continued until the pulses were tender (easily crushed with light pressure between the molars), and no raw taste was perceived as determined by a sensory evaluation expert experienced in tasting pulses. Cooked pulses were drained, rinsed with cold water, and refrigerated (no longer than 24 h) before incorporation with the food formulation.

Five different formulations were selected based on types of foods consumed containing beans and peas noted in the introduction. Using pulses prepared in formats most commonly consumed would increase the likelihood that participants would be willing to volunteer for the studies and comply with the conditions of the clinical trial. An additional consideration was their suitability for preparing in advance and freezing so that they could be distributed to clinical trial participants to take home frozen, and be reheated as required. Hence, salads were not chosen. The five formulations—Vegetable Soup (VS), Hamburger Soup (HS), Tortellini Soup (TS), Chicken Casserole (CC), and Zucchini Casserole (ZC)—are shown in Table 1 with the corresponding bean and pea type(s) that were added to each one. Thus, the only difference in the five formulations was the type of bean(s) or pea(s) that was added. For the clinical trial, batch sizes of 30 portions for each formulation were made at one time. Single portion sizes were determined by dividing the total cooked, cooled weight by 30. This single portion was placed into a medium-sized (17.7 × 18.8 cm) freezer bag (Ziploc, S.C. Johnson and Son, Limited, Brantford, ON, Canada) with the 120 g of cooked pulse which was either a single pulse type for VS, TS, CC, and ZC, or both types of peas (60 g yellow and 60 g green) for HS, or all four types of beans (40 g pinto, 40 g navy, 40 g black, and 40 g great northern) for HS. The rationale for the mixed pulse combination for HS is that there were four bean types and five dishes were required so that the clinical trial participants would consume a three-quarter cup serving of pulses five times per week. Hence, four of the dishes were made with one bean type and the fifth dish was made with a mixture of all four bean types, ensuring that all bean types were equally represented across the week. The same rationale applies for peas. There were two pea types so that two dishes contained green peas, two had yellow peas and one had a combination of green and yellow peas. Final cooked portion sizes as well as the proportion of pulses they contained are shown in Table 1. Samples were frozen (−20 °C) until required. For the consumer acceptability study, a batch of 30 portions was made for each of the five formulations along with 30 portions of their respective bean and pea types using the same methods described for the clinical trial foods. These were combined and a suitable amount for preparation for the consumer panelists (325 g) was placed into the same type of freezer bags as for the clinical trial and also frozen until required. The nutrient content (as-is basis) of the pulse-fortified foods (analyzed by Silliker, Canada Co., Markham, ON, Canada) is provided in Table 2.

### 2.3. Sample Preparation and Presentation for Sensory Evaluation

Samples for sensory evaluation by the consumer group were removed from the freezer approximately 24 h prior to the sensory evaluation and placed in the refrigerator (4 °C) to thaw. For heating, 325 g were placed into a 2.5 L stainless steel saucepan. The element range top (Frigidaire Household Electric Range, ES510 Control, Electrolux Canada Corp., Mississauga, ON, Canada) was set at moderate to high. Samples were stirred frequently to ensure even heating. Heating continued until the temperature of the more dense ingredients (for example, meat, beans, and tortellini) reached and was held at 85 °C for 2 min. Approximately 35 to 40 g were portioned into 125 mL Styrofoam cups labeled with 3-digit random numbers, capped, and placed into thermal bags to keep warm. Cups were placed on trays and passed through the front partition to consumers once they were seated at the work station. The temperature of foods for evaluation by the consumer panel participants was approximately 60 °C.

Clinical trial participants were instructed to remove one package of study food from the freezer and defrost it in the refrigerator. Once defrosted, it was to be heated before consumption. All of the food from the package was to be eaten at either lunch or supper. In order to meet personal preference, additional seasonings were allowed.

### 2.4. Sensory Methods

#### 2.4.1. Consumer Acceptability Study

##### Recruitment

For the consumer study, volunteers were recruited from the staff and student populations according to procedures approved by the Human Ethics Research Board at the University of Manitoba (Protocol # J2012:060). The only criteria were that volunteers were not allergic to any of the food products and that they be available and interested in the study. They were untrained. An honorarium was provided for their participation. As noted by Stone and Sidel [20], in order to detect a significant difference at the 0.05 level of probability, a standard deviation of 1.5 is required with 40 participants. The authors caution, however, that inherent variability within a sample such as different harvest dates or non-mass-produced product, as in this case, may necessitate larger sample sizes. This point is reinforced by Mammasse and Schlich [21], who found that panel sizes from 20 to 150 were adequate with the variability in numbers attributed to the level of complexity for the product space. Order effects can also be reduced with larger numbers of participants [20].

##### Sample Evaluation

Consumers were seated in individual partitioned work stations equipped with computerised sensory software (Sensory Integrated Management Systems, Morristown, NJ, USA 2011). Overhead fluorescent lighting was used. Filtered water was available for cleansing the palate as required. All consumers evaluated the five bean or five pea samples during one session in perfectly balanced randomised complete block order according to the sensory software program. Consumers were required to come to two sessions on two separate days to complete the study with all samples of one pulse type (bean or pea) presented at each session. Sessions were approximately one week apart. After smelling, observing, and tasting as much of the sample as desired, consumers rated the aroma, appearance, flavor, and texture as well as overall acceptance of the samples on 9-point hedonic scales where 9 = like extremely; 8 = like very much; 7 = like moderately; 6 = like slightly; 5 = neither like nor dislike; 4 = dislike slightly; 3 = dislike moderately; 2 = dislike very much; 1 = dislike extremely. The Food Action (FACT) rating scale [22] was used as another measure of acceptance based on how frequently consumers would eat the samples that they tasted. One of the following nine categories could be selected where 9 = I would eat this every opportunity I had; 8 = I would eat this very often; 7 = I would frequently eat this; 6 = I like this and would eat it now and then; 5 = I would eat this if available but would not go out of my way; 4 = I don’t like this but would eat it on an occasion; 3 = I would hardly ever eat this; 2 = I would eat this if there were no other food choices; 1 = I would eat this only if forced. Information was collected regarding gender, age, and how often peas and beans (pulses) were eaten.

#### 2.4.2. Clinical Trial Participants—Acceptability

A clinical trial (Clinical Trials.gov Identifier: NCT01661543) was designed to study the effect of the consumption of beans and peas by healthy women and men with slightly elevated cholesterol level, deemed to be when LDL-cholesterol was between 3.00 mmol/L and 5.00 mmol/L. Within this range the cholesterol is high enough that a measurable effect on cholesterol lowering can be detected with dietary intervention but low enough that the participant is not taking medications or using alternative measures to lower cholesterol. It was hypothesised that blood lipid profiles would improve significantly. Subjects in the trial consumed food portions that contained peas or beans in an amount equal to approximately ¾ cup per serving five times during the week for a total of 3.5 cups of cooked pulses per week.

Clinical trial participants were recruited from the local community according to approved protocols at both the Edmonton (University of Alberta) and Winnipeg (CCARM) sites. The clinical trial sample size was powered to detect a significant effect (0.5 standard deviations) on the primary outcome measure of LDL-cholesterol with 80% power. All participants provided written informed consent prior to any involvement in the study. Participants were randomly assigned to one of three study arms consuming foods containing peas, beans, or rice (pulse-free comparator for the clinical trial). They were instructed to eat each formulation once a week for six weeks. Study visits to the clinic were scheduled for blood and urine collection at 0, 3, and 6 weeks. At the last study visit, participants for the clinical trial at both sites were instructed to complete the same questionnaire as the consumer group regarding the acceptability of the study foods that they had eaten. No training was given. An honorarium was provided for their participation.

#### 2.4.3. Statistical Analysis

For each group, two-way linear analysis of variance was conducted with the model containing pulse type and sample as main fixed effects and the pulse by sample interaction. When the interaction was not significant, the sums of squares were pooled with the error term as recommended by O’Mahony [23]. F values were recalculated with the additional sums of squares for error and the corresponding degrees of freedom. Tukey’s multiple comparison test was used to determine the mean treatment differences when significant (*p* < 0.05). SAS (2009) software (Statistical Analysis System, Cary, NC, USA) was used for the analysis. To determine a possible association between nutrients (for example, total dietary fibre, insoluble fibre, omega 3 and 6 fatty acids, iron, protein, Calories, and folate) and the acceptability of the foods, partial least squares (PLS) regression was performed (XLSTAT, Addinsoft) using overall mean values for acceptability of the study foods by the consumer group and also by the clinical trial group. Two factors were considered, and the Jackknife method was the validation technique used. For the clinical trial group, cumulative Q^2^, R^2^X, and R^2^Y values were 0.441, 0.856, and 0.512, respectively. For the consumer group, cumulative Q^2^, R^2^X, and R^2^Y values were 0.135, 0.680, and 0.522, respectively. Variable importance in the projection (VIP) scores for the PLS regression that are greater than 1 are highly influential. For the clinical trial group these included starch, sucrose, beta carotene, total sugar, sodium, saturated fat, trans fatty acid, soluble dietary fibre, and glucose for Component 1, and the same variables plus cholesterol for Component 2. For the consumer group, VIP scores greater than 1 were found for sodium, sucrose, total sugar, glucose, monounsaturated fat, starch, beta carotene, total fat, and saturated fat for Component 1. The same variables had VIP scores greater than 1 and also included CaloriesUSA for Component 2. Internal preference mapping (XLSTAT, Addinsoft), a statistical method based on principal component analysis (PCA), was used to identify numbers of consumers’ responses for overall acceptability corresponding to the food samples. For visual interpretation the consumers are retained on a virtual circle surrounding the product points. Two components were considered for this analysis which was done using standardised values and correlations. Eigenvalues for the clinical trial group were 16.254 and 12.672 for Components 1 and 2, respectively, and for the consumer group were 37.112 and 23.655 for Components 1 and 2, respectively.

## 3. Results and Discussion

### 3.1. Nutrients

Insoluble dietary fibre was higher in all of the samples compared to the soluble fibre (Table 2), which agrees with results of analyses from previous studies [1]. Study foods containing beans had more fibre than those containing peas due to the higher overall fibre in beans compared with peas [1]. Bean samples in the present study were higher in folate for all of the study foods except for ZC where the bean and pea samples were similar. This could be due to the pasta ingredient in this dish which is enriched with folic acid, resulting in an increase in the amount for the pea sample to make it more similar to the bean sample. Jha and co-workers [24] also found that the folate content of beans was higher than that of pea cultivars. Folate is an important mineral for prevention of neural tube defects, and it contributes to normal cognitive function and cardiovascular health [25]. Minerals were also generally higher in bean samples than pea samples.

### 3.2. Demographics

Participants in the consumer study were the same for the samples containing peas and beans except for two volunteers who were not available for tasting the samples containing peas, thus making the total number of consumers for bean tasting 110 and for pea tasting 108. The clinical trial group consisted of 59 participants for the bean-containing foods (34 from Alberta and 25 from Manitoba) and 58 participants for the pea-containing foods (33 from Alberta and 25 from Manitoba). The distribution of participants in the two groups in terms of gender and age is shown in Table 3. Females dominated the groups ranging from 71% of the sample for the consumer group to about 68% for the clinical trial group. Ages from the consumer group were represented in all categories from age 18 to 65 years and over with 73% of the group 44 years and younger. The university serves a clientele that for the most part is found within this age range. On the other hand, clinical trial participants were represented by the older age groups and 85% of them were 45 years and above. This is to be somewhat expected as criteria for inclusion in the study were that participants have elevated cholesterol levels (to be able to respond to the dietary intervention) but not high enough to be taking cholesterol-lowering medications or to be adhering to other measures to lower cholesterol. Thus, older-aged participants were more likely to fit the recruitment criteria in the clinical trial. Regarding the frequency of eating peas and beans (pulses), for the consumer group, it was found that “two to three times a week” and “at least once a week” were the frequencies noted by the largest number of respondents. “At least once a month” or less often were categories given by about 15% of the consumers in the study. For the clinical trial participants, because of the exclusion criteria, those eligible for the clinical trial had to be consuming two or fewer servings of pulses per week.

### 3.3. Sample Evaluation

The overall objective of this research was to determine the acceptability of foods containing peas and beans which would provide information in the future for eventual commercialisation of the formulations. Since it is possible that the acceptability of the formulations may be influenced by the type of pulse that is added, the interaction of pulse by sample was tested. For the consumer study, a significant interaction (*p* < 0.05) was found for appearance, texture, overall acceptability, and FACT (Table 4). Interaction plots determined that for appearance, the sample containing peas was higher in acceptability for the HS, VS, and ZC formulations, whereas for CC and TS, the sample containing beans had higher acceptability. For texture, overall acceptability, and FACT, bean samples were higher in acceptability for CC, HS, and TS, whereas for VS and ZC, pea samples were higher in acceptability. Significant interaction was not found for acceptability of aroma and flavor. For aroma, CC (mean value (MV) = 6.8) was significantly higher in acceptability (*p* < 0.05) than ZC (MV = 6.4). The flavor acceptability of CC (MV = 7.2) was also significantly higher than that of ZC (MV = 4.9), as well as significantly higher than that of HS (MV = 6.8). FACT results showed that VS (MV = 6.3) was significantly higher than HS (MV = 5.8), which in turn was significantly higher than ZC (MV = 4.2). Overall, MVs for all of the attributes for all dishes except for ZC ranged from 6.0 (like slightly) to 7.2 (like moderately). The range of MVs for ZC was from 4.9 (neither like nor dislike) to 6.4 (like slightly). FACT MVs ranged from 6.3 to 5.8 (I like this and would eat it now and then) for all dishes except for ZC which had a MV of 4.2 (I don’t like this but would eat it on occasion). No significant difference was found between foods made with peas and beans.

Results from the clinical trial, however, did not follow these same trends. No significant interaction was found for any of the attributes (Table 4). Unlike the consumer study, pulse type showed that beans had significantly higher acceptability for appearance, texture, and overall acceptability. VS (MV = 7.2 to 6.7) and HS (MV = 7.2 to 6.6) were significantly higher in acceptance for all attributes than TS (MV = 6.3 to 5.7) and ZC (MV = 6.0 to 5.4). ZC (MV = 5.4) was significantly lower in acceptability for all of the attributes except for texture of TS (MV = 5.7). Results for FACT were similar to those found for the consumer group except the MV for ZC was slightly higher at 4.6. These results showed that no particular food was preferred by both groups but that each group had their own preferences. Factors influencing this could be the younger age of the consumer group, the fact they only consumed the product once, the environment of the test site which was not their usual home setting, the lack of opportunity to add seasonings that they might typically use to enhance the palatability of the foods, and that their participation was not health motivated.

Overall, the MVs for the formulations for acceptability of all of the attributes ranged from 4.9 (neither like nor dislike) to 7.2 (like moderately). These values are similar to those found by a consumer acceptance panel for lentil-fortified soups and casserole dishes which ranged from 5.0 to 7.0 [18]. Clinical trial interventions consisting of a variety of food types containing a variety of pulse types were scored from 6.4 to 7.5 on a 9-point scale from 1—extremely unpleasant to 9—extremely pleasant [26]. These ratings are somewhat similar to those found in the current study. Consumer liking attributes for beans included sweet taste, soft texture, and cooked-bean flavors [27]. CC was among the samples with the highest mean acceptability values which may be attributed to the fact that the bean proportion was highest of all of the samples (48%) (Table 1).

### 3.4. Regression Analysis

**Figure 1:**
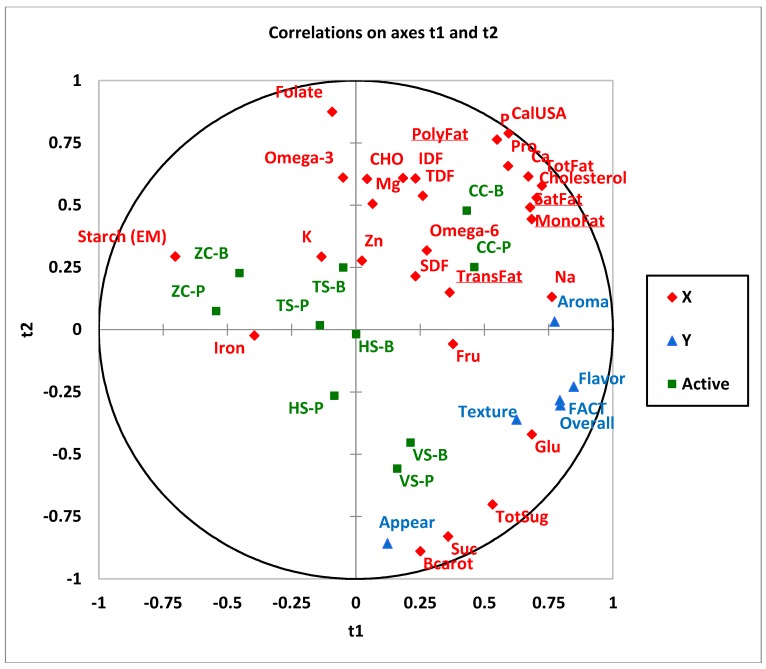
Correlation of sensory acceptability for the consumer group and selected nutrients—10 study foods. Partial least square (PLS) correlation loadings for X (t1) and Y (t2), where X variables 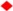 are nutrients and Y variables 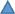 are acceptability measurements including appearance, aroma, flavor, texture, overall acceptability, and FACT (frequency of eating the sample). 10 samples (Active) 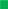 TS-B, Tortellini Soup Bean; TS-P, Tortellini Soup Pea; VS-B, Vegetable Soup Bean; VS-P, Vegetable Soup Pea; ZC-B, Zucchini Casserole Bean; ZC-P, Zucchini Casserole Pea; CC-B, Chicken Casserole Bean; CC-P, Chicken Casserole Pea; HS-B, Hamburger Soup Bean; HS-P, Hamburger Soup Pea. Abbreviations: IDF, insoluble dietary fibre; Mg, magnesium; CalUSA, Calories USA; TotFat, Total Fat; SatFat, Saturated Fat; MonoFat, Monounsaturated Fat; PolyFat, Polyunsaturated Fat; Na, Sodium; K, Potassium; CHO, Carbohydrate; TDF, Total Dietary Fibre; TotSug, Total Sugar; Fru, Fructose; Glu, Glucose; Suc, Sucrose; Bcarot, Beta Carotene; Ca, Calcium; P, Phosphorous; SDF, Soluble Dietary Fibre; Zn, Zinc.

Results from PLS regression analysis established the associations between the acceptability of the study foods containing peas and beans and the selected nutrients that they contain. For the consumer group, appearance, texture, flavor, overall acceptability, and FACT are found throughout the bottom-right section of the correlation loading plot (Figure 1), sharing space with the nutrients glucose, total sugar, sucrose, and beta carotene. VS-P and VS-B are the samples in this area suggesting that VS with either beans or peas are the most acceptable samples, possibly due to their sugar content. Beta carotene is in close proximity to appearance indicating that color is positively influencing the acceptability of the appearance. Aroma appears just above the *x* axis in the top-right section. Also in this section are the majority of the nutrients including fatty acids, fibre, cholesterol, minerals (sodium, zinc, magnesium, calcium, phosphorous), protein and Calories. The only sample in this section is CC with both peas and beans. These samples are in the same half with the acceptability attributes and hence would exhibit higher acceptability than the ZC-B and ZC-P which are found opposite the acceptability attributes. TS-P and TS-B would be considered closer in acceptability to the VS and HS-P, and HS-B even closer in acceptability to VS.

**Figure 2:**
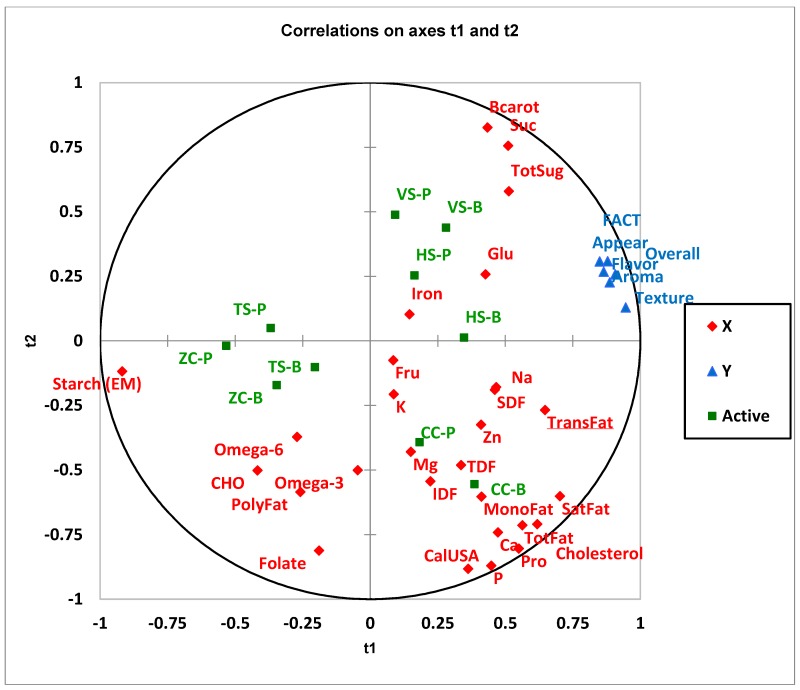
Correlation of sensory acceptability for the clinical trial group and selected nutrients—10 study foods. Partial least square (PLS) correlation loadings for X (t1) and Y (t2), where X variables 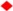 are nutrients and Y variables 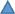 are acceptability measurements including appearance, aroma, flavor, texture, overall acceptability, and FACT (frequency of eating the sample). 10 samples (Active) 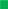 TS-B, Tortellini Soup Bean; TS-P, Tortellini Soup Pea; VS-B, Vegetable Soup Bean; VS-P, Vegetable Soup Pea; ZC-B, Zucchini Casserole Bean; ZC-P, Zucchini Casserole Pea; CC-B, Chicken Casserole Bean; CC-P, Chicken Casserole Pea; HS-B, Hamburger Soup Bean; HS-P, Hamburger Soup Pea. Abbreviations: IDF, insoluble dietary fibre; Mg, magnesium; CalUSA, Calories USA; TotFat, Total Fat; SatFat, Saturated Fat; MonoFat, Monounsaturated Fat; PolyFat, Polyunsaturated Fat; Na, Sodium; K, Potassium; CHO, Carbohydrate; TDF, Total Dietary Fibre; TotSug, Total Sugar; Fru, Fructose; Glu, Glucose; Suc, Sucrose; Bcarot, Beta Carotene; Ca, Calcium; P, Phosphorous; SDF, Soluble Dietary Fibre; Zn, Zinc.

Similar results were found for the clinical trial group in terms of groupings of the samples (Figure 2). However, unlike the consumer group, the acceptability attributes are very close to one another. This indicates that the clinical trial group does not appear to be as discriminating in terms of the acceptability of specific sensory attributes. Questionnaires were completed by the clinical trial participants during their last study visit but unlike the consumer panel the samples were not presented at this time. This could be a reason why their discrimination between the attributes was less pronounced. However, they had been eating the foods once a week for six weeks so if an attribute was unacceptable this would likely be reflected in the values. Both the VS and HS containing peas and beans share the same quadrant with the acceptability parameters. In addition to the sugars and beta carotene as shown in Figure 1 for the VS, iron is also included due to the proximity of HS. The clinical trial participants had a similar separation of the CC-P and CC-B samples from the ZC-P, ZC-B, TS-P, and TS-B as was observed for the consumer group.

It appears from this correlation analysis that the nutrient content is influenced to a greater degree by the formulation rather than the pulse type as the pea and bean versions of the same formulation are relatively close to one another. Fibre is found in close proximity to CC-P and CC-B, possibly due to the higher percentage of pulse in this sample compared with the others (48% vs. 36.6 ± 2.4% (standard deviation) (Table 1). The CC sample with beans and peas is also associated with other nutrients such as fat, protein, carbohydrate, and minerals.

**Figure 3:**
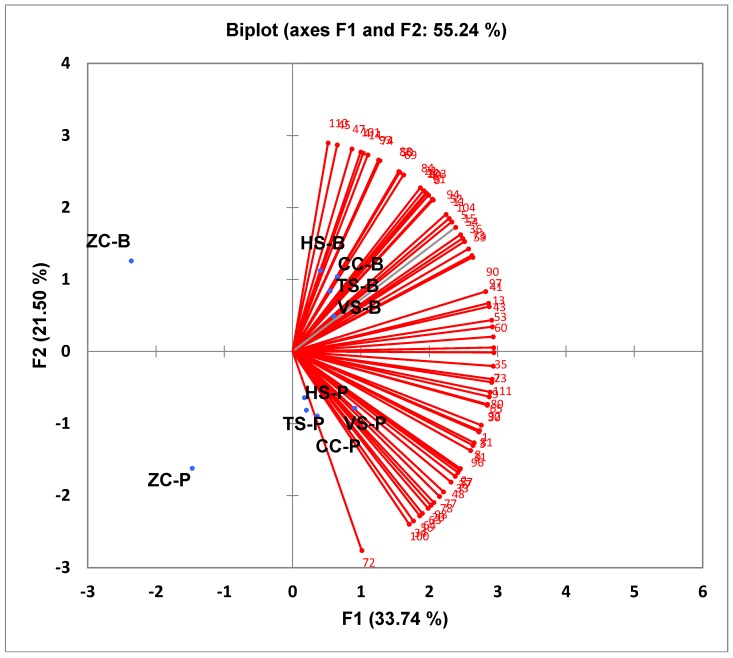
Internal preference map for overall acceptability for the consumer group—10 study foods. TS-B, Tortellini Soup Bean; TS-P, Tortellini Soup Pea; VS-B, Vegetable Soup Bean; VS-P, Vegetable Soup Pea; ZC-B, Zucchini Casserole Bean; ZC-P, Zucchini Casserole Pea; CC-B, Chicken Casserole Bean; CC-P, Chicken Casserole Pea; HS-B, Hamburger Soup Bean; HS-P, Hamburger Soup Pea.

Overall acceptance of the five study foods containing both peas and beans for the consumer group is shown in Figure 3. Acceptance values for each member of the consumer group are plotted and the 10 samples are distributed in the space accordingly. The top portion of the right side of the biplot contains the bean samples for HS, CC, TS, and VS. The bottom portion contains the same samples with peas. This indicates that there is separation between the pea and bean samples, but they are all receiving positive responses. The far-left side of the graph has no responses and contains ZC with the bean sample at the top as with the right side and the pea sample at the bottom. Therefore, the samples accepted by the majority of respondents are both pea- and bean-containing HS, CC, TS, and VS.

**Figure 4:**
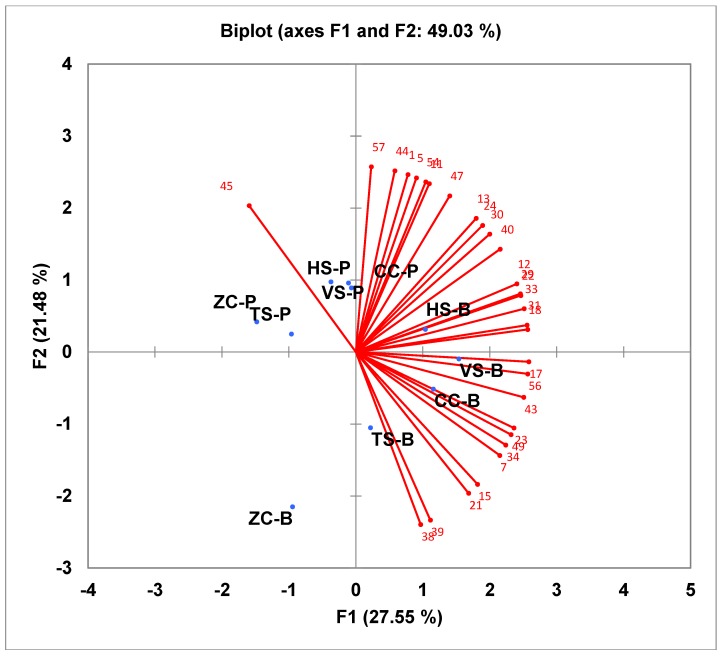
Internal preference map for overall acceptability for the clinical trial group—10 study foods. TS-B, Tortellini Soup Bean; TS-P, Tortellini Soup Pea; VS-B, Vegetable Soup Bean; VS-P, Vegetable Soup Pea; ZC-B, Zucchini Casserole Bean; ZC-P, Zucchini Casserole Pea; CC-B, Chicken Casserole Bean; CC-P, Chicken Casserole Pea; HS-B, Hamburger Soup Bean; HS-P, Hamburger Soup Pea.

Figure 4 shows the overall acceptance results for the same samples for the clinical trial group. Similar to the results from the consumer group, the responses of the participants are shown radiating from the centre toward the right side of the graph. The samples are more spread throughout the area. The bean samples are found on the bottom-right side of the figure except for ZC-B which is in the bottom-left quadrant. The pea samples are found in both quadrants above the *x* axis with CC on the right, HS and VS in the middle, and ZC and TS on the left. As there are no responses for ZC-P, TS-P, and ZC-B, these samples are least liked of the 10 samples. Separation of the samples was more distinct for the consumer group, yielding four clusters: HS, CC, TS, and VS with beans; HS, CC, TS, and VS with peas; ZC-B; and ZC-P. Obvious clusters were not shown for the clinical trial group except for ZC-B which is on its own.

## 4. Conclusions

In summary, acceptability of the five formulations made with peas and beans for these two groups differed in some respects. The consumer group showed some preferences for pulse type depending on the formulation whereas the clinical trial group did not. The clinical trial group liked beans more than peas for appearance, texture, and overall acceptability attributes. Generally, the clinical trial group preferred VS and HS whereas the consumer group preferred CC. Influential factors could include motivation for healthy eating, age, number of times the product was consumed, amount of the product consumed, and where it was consumed. Further studies are needed to investigate their possible impact. In the future, it would be advised to have clinical trial participants evaluate both bean and pea formulations before the trial began and also after the trial finished in a similar setting to the consumer panel, so that statistically valid comparisons could be made. PLS regression showed that the clinical trial participants were less discriminating than the consumer participants in their evaluation of the acceptability of the different sensory attributes. This provides evidence that acceptability testing needs to be done for both groups in order to obtain information regarding acceptability of foods for specific attributes. ZC was found to be the lowest in acceptance for both groups and particularly the bean sample for the clinical trial group. CC with beans had a high acceptability overall, contained a large proportion of pulses, provided the best nutrient profile, and thus would be a good candidate for commercialisation.

## Figures and Tables

**Table 1:** Formulations (1 portion) for five study foods including the cooking methods ^1^.

	Study Food Formulation Ingredients-Amounts for 1 Portion									
Food Item and Pulse Type	Zucchini Casserole—Navy Bean, or Yellow Pea		Vegetable Soup—Black Bean, or Yellow Pea		Chicken Casserole—Great Northern Bean, or Green Pea		Tortellini Soup—Pinto Bean, or Green Pea		Hamburger Soup—All Four Bean Types, or Both Pea Types	
**Study Food Raw Ingredients**	g	% (*w/w*)	g	% (*w/w*)	g	% (*w/w*)	g	% (*w/w*)	g	% (*w/w*)
Black pepper			0.1	0.03	0.03	0.02				
Canola oil	1.0	0.4	1.0	0.3	1.0	0.6	1.0	0.5		
Carrot, fresh, sliced			25.0	8.4						
Chicken powder, 25% less salt			4.5	1.5	4.5	2.7				
Chicken, thigh, boneless, skinless					40.0	23.9				
Corn, niblets, frozen			45.0	15.1						
Cumin, dried	0.03	0.01	0.03	0.01	0.03	0.02	0.03	0.01	0.03	0.01
Garlic, minced	4.0	1.7	3.0	1.0	3.0	1.8	3.0	1.4	3.0	1.2
Green pepper, fresh, chopped					30.0	17.9	20.0	9.4		
Ground beef, lean									40.0	15.5
Italian seasoning							0.2	0.09		
Mixed vegetables									40.0	15.5
Mozzarella cheese, part-skim (18% fat)					10.0	6.0				
Mushrooms, white, fresh, sliced	25.0	10.6					20.0	9.4		
Onion soup, dried									4.5	1.8
Onion, fresh large yellow, diced	30.0	12.7	50.0	16.7	30.0	17.9	25.0	11.7	20.0	7.8
Oregano, leaves, dried	0.16	0.07			0.1	0.06				
Parsley, dried			0.1	0.03						
Pasta, baby shells	15.0	6.4								
Red pepper, sweet, fresh, chopped					30.0	17.9	20.0	9.4		
Salt	0.06	0.03								
Summer savory, dried			0.1	0.03						
Thyme, dried leaves	0.12	0.05	0.1	0.03						
Tomato sauce, low sodium			20.0	6.7					10.0	3.9
Tomatoes, canned, diced, Italian style									40.0	15.5
Tomatoes, canned, diced	60.0	25.5	70.0	23.4						
Tortellini, rainbow, three cheese							20.0	9.4		
Vegetable powder							4.5	2.1		
Water	60.0	25.5	80.0	26.8	13.0	7.8	80.0	37.4	100.0	38.8
Yogurt, low fat (1.6%), plain					6.0	3.6				
Zucchini, fresh, diced	40.0	17.0					20.0	9.4		
**Study Food Raw Ingredients—Total Weight**	235.4		298.9		167.7		213.7		257.5	
**Pulse—Cooked Weight**	120.0		120.0		120.0		120.0		120.0	
**Study Food—Total Cooked Weight**	325		360		251		307		323	
**Pulse—% of Cooked Study Food**	36.9		33.3		47.8		39.1		37.2	

**^1^Cooking Methods: Zucchini Casserole.** Heat oil with onion and garlic on low in saucepan for about 4 min or until onion is translucent. Stir occasionally. Cut mushrooms into quarters. Cut zucchini lengthwise in quarters then into 1–1.5 cm thick slices. Add mushrooms and zucchini; cook for 2 min or until softened. Stir in tomatoes, salt, pasta, thyme, oregano, and water. Bring to a boil; reduce heat and simmer covered for 10 min or until pasta is tender but still firm. Ensure that an internal temperature of 65 °C is reached. Cool at 4 °C for no longer than 24 h. Combine with cooked, cooled yellow peas (120 g) or navy beans (120 g). **Vegetable Soup** Heat oil with onion and garlic on low in saucepan for about 4 min or until onion is translucent. Stir occasionally. Stir in carrot and corn and cook another 2–3 min. Combine chicken powder and water and add to saucepan. Add tomatoes, thyme, savory, parsley, pepper, and cumin and bring to a boil. Reduce heat to a simmer, uncovered, and cook approximately 30 min. Ensure that an internal temperature of 65 °C is reached. Cool at 4 °C for no longer than 24 h. Combine with cooked, cooled yellow peas (120 g) or black beans (120 g). **Chicken Casserole** Heat oil with onion and garlic on low in saucepan for about 4 min or until onion is translucent. Stir occasionally. Add chicken, increase heat to medium and cook until chicken reaches an internal temperature of 75 °C. Add green and red pepper and cook until softened. Add chicken powder to water and dry seasonings and mix well. Reduce heat and simmer covered for 10–15 min. Add yogurt. Add cheese slowly while stirring. Ensure that an internal temperature of 65 °C is reached. Cool at 4 °C for no longer than 24 h. Combine with cooked cooled green peas (120 g) or great northern beans (120 g). **Tortellini Soup** Heat oil with onion and garlic on low in saucepan for about 4 min or until onion is translucent. Stir occasionally. Stir in mushrooms, peppers, and zucchini and cook another 2–3 min until vegetables are softened. Combine vegetable powder and water and add to saucepan. Add seasoning and bring to a boil. Add tortellini. Reduce heat to a simmer, cover, and cook approximately 5–7 min. Ensure that an internal temperature of 65 °C is reached. Cool at 4 °C for no longer than 24 h. Combine with cooked cooled green peas (120 g) or pinto beans (120 g). **Hamburger Soup** Cook onion and garlic on low in saucepan for about 4 min or until onion is translucent. Stir occasionally. Add beef, increase heat to medium and cook until beef reaches an internal temperature of 75 °C. Drain juices. Add tomatoes, tomato sauce, vegetables, dried onion soup, cumin, and water; bring to a boil. Reduce heat and simmer covered for 30 min. Ensure that an internal temperature of 65 °C is reached. Cool at 4 °C for no longer than 24 h. Combine with cooked cooled yellow peas (60 g) and green peas (60 g) or navy beans (40 g), black beans (40 g), pinto beans (40 g), and great northern beans (40 g).

**Table 2:** Nutrients per serving contained in study foods for samples containing peas and beans.

Nutrient	Unit	Zucchini Casserole		Vegetable Soup		Chicken Casserole		Tortellini Soup		Hamburger Soup		Method of Analysis
		Navy Bean	Yellow Pea	Black Bean	Yellow Pea	Great Northern Bean	Green Pea	Pinto Bean	Green Pea	4 Bean Types	2 Pea Types	
Serving size	g	325	325	360	360	251	251	307	307	323	323	
Energy	Cal USA	283	263	288	281	279	237	295	270	294	275	Atwater Factors
Energy	Cal Canada	244	237	248	252	241	215	246	246	252	242	Health Canada 20
Total fat as triglycerides by GC	g	3.19	2.93	3.71	3.53	4.64	4.19	3.96	3.96	4.91	3.97	AOAC 996.06
Saturated fatty acids	g	0.59	0.49	0.79	0.68	1.68	1.48	0.83	0.80	1.91	1.42	
Cis-monounsaturated fatty acids	g	0.88	0.91	1.12	1.19	1.38	1.42	1.35	1.57	1.52	1.29	
Cis-polyunsaturated fatty acids	g	1.56	1.37	1.55	1.44	1.28	1.03	1.54	1.38	1.03	0.94	
Omega-6 fatty acids	g	1.04	1.14	1.15	1.26	0.90	0.88	1.04	1.07	0.68	0.78	
Omega-3 fatty acids	g	0.52	0.23	0.40	0.18	0.38	0.15	0.49	0.28	0.36	0.16	
Trans fatty acids	g	<0.01	<0.01	<0.01	<0.01	0.05	0.06	<0.01	<0.01	0.16	0.10	
Conjugated linoleic acid	g	<0.01	<0.01	<0.01	<0.01	<0.01	<0.01	<0.01	<0.01	<0.01	<0.01	
Cholesterol	mg	<1.0	<1.0	<1.0	<1.0	37.1	29.0	<1.0	<1.0	17.8	13.6	AOAC 994.19
Carbohydrate	g	44.2	45.8	47.5	47.9	36.1	31.0	49.1	44.8	41.0	40.7	Calculation
Soluble dietary fibre	g	2.6	1.3	4.7	0.7	4.0	0.4	3.4	0.6	2.6	3.2	AOAC 991.42; AOAC 985.29
Insoluble dietary fibre	g	16.6	12.4	15.8	14.0	14.6	10.3	20.9	12.0	18.4	12.3	AOAC 991.42
Total dietary fibre	g	19.2	13.7	20.5	14.8	18.6	10.8	24.3	12.6	21.0	15.5	AOAC 985.29
Total sugars	g	4.2	3.3	10.1	10.4	4.3	3.4	3.7	4.0	4.5	4.5	AOAC 982.14
Fructose	g	2.11	2.15	2.56	2.70	1.78	1.55	1.69	1.75	1.58	1.58	
Glucose	g	1.30	1.17	2.56	2.99	1.61	1.40	1.20	1.60	1.00	1.45	
Sucrose	g	0.81	<0.2	5.04	4.68	0.88	0.43	0.89	0.64	2.00	1.65	
Maltose	g	<0.5	<0.5	<0.5	<0.5	<0.5	<0.5	<0.5	<0.5	<0.5	<0.5	
Lactose	g	<0.5	<0.5	<0.5	<0.5	<0.5	<0.5	<0.5	<0.5	<0.5	<0.5	
Starch by enzymatic method	% (w/w)	11.57	13.23	6.80	7.56	5.20	4.95	10.59	14.92	6.69	7.78	AOAC 979.10
Protein (factor 6.25)	g	19.53	13.59	16.34	14.54	22.87	18.79	15.66	13.91	21.38	18.86	AOAC 992.15
Beta carotene	IU	455	510	4342	3139	259	288	350	473	1331	1596	AOAC 2001.13
Folate	mcg	88.7	82.6	37.4	26.3	100.1	34.0	83.8	56.5	58.5	26.8	AOAC 944.12
Calcium	mg	124.2	88.1	144.7	102.6	172.7	118.7	112.1	85.7	107.6	70.7	AOAC 984.27
Iron	mg	5.2	3.9	4.7	3.6	3.0	1.6	3.7	3.1	6.1	4.5	AOAC 984.27
Magnesium	mg	96.5	78.7	105.1	75.2	84.1	46.0	95.2	70.6	91.4	65.9	AOAC 984.27
Phosphorus	mg	295.4	225.9	275.0	202.3	366.5	253.7	259.1	213.1	299.1	216.4	AOAC 984.27
Potassium	mg	874	640	860	644	725	234	728	531	788	497	AOAC 984.27
Sodium	mg	160.9	156.7	676.8	615.6	625.0	438.6	718.4	724.5	513.6	426.4	AOAC 984.27
Zinc	mg	2.5	2.7	2.2	2.3	2.7	1.6	2.1	2.1	3.6	3.5	AOAC 984.27
Moisture	g	255.2	260.2	288.1	290.5	183.4	158.0	234.2	240.8	252.0	256.6	AOAC 964.22 Soups; 950.46Ba Casseroles
Ash	g	2.73	2.41	4.18	3.49	3.89	2.97	4.11	3.53	3.59	2.97	AOAC 920.153

**Table 3:** Number (followed in brackets by percentage) for age and gender of participants in consumer and clinical trial groups for acceptability of foods containing beans or peas.

	Foods Containing Beans		Foods Containing Peas	
	Consumer	Clinical Trial	Consumer	Clinical Trial
*Gender*				
Female	78 (70.9)	41 (69.5)	77 (71.3)	39 (67.2)
Male	32 (29.1)	18 (30.5)	31 (28.7)	19 (32.8)
*Age*				
18–24 years	37 (33.6)	0	36 (33.3)	0
25–34 years	28 (25.5)	4 (6.8)	28 (25.9)	5 (8.6)
35–44 years	15 (13.6)	5 (8.5)	14 (13.0)	4 (6.9)
45–54 years	14 (12.7)	12 (20.3)	14 (13.0)	16 (27.6)
55–64 years	11 (10.0)	22 (37.3)	11 (10.2)	22 (37.9)
65 years and over	5 (4.5)	16 (27.1)	5 (4.6)	11 (19.0)
*Total Number*	110	59	108	58

**Table 4:** Mean values (followed by the standard deviations in brackets) for sample and pulse type for consumer acceptability of study foods containing beans or peas by two groups—consumer and clinical trial participants—and *F* values.

	Consumer Participants										Clinical Trial Participants									
	Sample					Pulse Type		*F* Values			Sample					Pulse Type		*F* Values		
Attribute	ZC ^2^	VS	CC	TS	HS	Bean	Pea	Sample (S)	Pulse (P)	P × S	ZC	VS	CC	TS	HS	Bean	Pea	Sample (S)	Pulse (P)	P × S
Aroma ^3^	6.4 ^b^ (1.5)	6.7 ^ab^ (1.5)	6.8 ^a^ (1.5)	6.7 ^ab^ (1.6)	6.5 ^ab^ (1.5)	6.7 (1.5)	6.6 (1.5)	3.23 *	0.98 NS **^1^**	†	5.6 ^c^ (1.8)	7.0 ^a^ (1.5)	6.9 ^ab^ (1.6)	6.3 ^b^ (1.6)	7.0 ^a^ (1.5)	6.6 (1.7)	6.5 (1.7)	16.83 ***	0.36 NS	†
Appearance ^3^	6.2 ^bc^ (1.6)	7.1 ^a^ (1.4)	6.0 ^c^ (1.7)	5.9 ^c^ (1.7)	6.5 ^b^ (1.5)	6.3 (1.7)	6.4 (1.6)	18.78 ***	0.92 NS	2.81 *	6.0 ^c^ (1.7)	7.1^a^ (1.4)	6.6 ^ab^ (1.6)	6.3 ^bc^ (1.7)	7.0 ^a^ (1.5)	6.8 ^a^ (1.6)	6.4 ^b^ (1.7)	10.43 ***	12.92 ***	†
Flavor ^3^	4.9 ^c^ (1.8)	7.2 ^ab^ (1.4)	7.2 ^a^ (1.3)	6.9 ^ab^ (1.5)	6.8 ^b^ (1.6)	6.5 (1.8)	6.7 (1.7)	85.36 ***	1.71 NS	†	5.4 ^c^ (2.0)	7.1^a^ (1.7)	6.9 ^a^ (1.7)	6.3 ^b^ (1.8)	7.3 ^a^ (1.6)	6.6 (2.0)	6.6 (1.8)	22.74 ***	0.08 NS	†
Texture ^3^	5.3 ^b^ (1.8)	6.9 ^a^ (1.5)	6.5 ^a^ (1.8)	6.7 ^a^ (1.5)	6.7 ^a^ (1.6)	6.4 (1.8)	6.4 (1.6)	35.39 ***	0.09 NS	8.90 ***	5.4 ^b^ (2.0)	6.7^a^ (1.7)	6.6 ^a^ (1.8)	5.7 ^b^ (1.8)	6.6 ^a^ (1.8)	6.5 ^a^ (1.8)	6.0 ^b^ (2.0)	12.83 ***	10.92 **	†
Overall Acceptability ^3^	4.9 ^c^ (1.7)	7.1 ^a^ (1.4)	6.8 ^ab^ (1.6)	6.7 ^ab^ (1.5)	6.6 ^b^ (1.6)	6.4 (1.8)	6.4 (1.7)	68.15 ***	0.03 NS	3.73 **	5.5 ^c^ (2.2)	7.2 ^a^ (1.5)	6.8 ^ab^ (1.9)	6.3 ^b^ (1.9)	7.2 ^a^ (1.6)	6.8 ^a^ (1.9)	6.4 ^b^ (1.9)	18.02 ***	7.18 **	†
FACT ^4^	4.2 ^c^ (1.7)	6.3 ^a^ (1.7)	6.0 ^ab^ (1.8)	6.0 ^ab^ (1.5)	5.8 ^b^ (1.7)	5.6 (1.9)	5.7 (1.8)	52.35 ***	0.07 NS	3.17 *	4.6 ^c^ (2.2)	6.5 ^a^ (1.7)	6.1 ^ab^ (1.9)	5.5 ^b^ (1.9)	6.4 ^a^ (1.8)	5.9 (2.1)	5.8 (2.0)	20.45 ***	0.25 NS	†

^1^ NS, Not Significant *p* ≥ 0.05, * *p* < 0.05, ** *p* < 0.01, *** *p* < 0.001. †—not significant in original model; therefore, sums of squares taken out of the model and pooled with the error. ^2^ ZC, Zucchini Casserole; VS, Vegetable Soup; CC, Chicken Casserole; TS, Tortellini Soup; HS, Hamburger Soup. ^3^ 9 = like extremely; 8 = like very much; 7 = like moderately; 6 = like slightly; 5 = neither like nor dislike; 4 = dislike slightly; 3 = dislike moderately; 2 = dislike very much; 1 = dislike extremely. ^4^ Food Action Rating Scale: 9 = I would eat this every opportunity I had; 8 = I would eat this very often; 7 = I would frequently eat this; 6 = I like this and would eat it now and then; 5 = I would eat this if available but would not go out of my way; 4 = I don’t like this but would eat it on occasion; 3 = I would hardly ever eat this; 2 = I would eat this if there were no other food choices; 1 = I would eat this only if forced. ^abc^ mean values followed by the same letter within the same row within the same variable (sample, pulse type) for each group are not significantly different when a probability level of *p* < 0.05 is applied.
